# A Novel Intervention Technology for Cerebral Palsy: Brain Stimulation

**Published:** 2019

**Authors:** Sara RAMEZANI, Naser AMIINI, Fatemeh KHODAEI, Hosein SAFAKHEIL, Arash SARVEAZAD, Seyedeh Lena MOHEBBI, Peiman BROUKI MILAN

**Affiliations:** 1 Neuroscience Research Center, School of Medicine, Guilan University of Medical Sciences, Rasht, Iran; 2 Guilan Road Trauma Research Center, Guilan University of Medical Sciences, Rasht, Iran; 3 Cellular and Molecular Research Center, Iran University of Medical Sciences, Tehran, Iran; 4 Institute of Regenerative Medicine, Faculty of Advanced Technologies in Medicine, Iran University of Medical Sciences, Tehran, Iran; 5 Burn Center, Iran University of Medical Sciences, Tehran, Iran; 6 Colorectal Research Center, Iran University of Medical Sciences, Tehran, Iran; 7 Department of Tissue Engineering & Regenerative Medicine, Iran University of Medical Sciences, Tehran, Iran

**Keywords:** Cerebral palsy, Brain stimulation, Pediatric disorder

## Abstract

A common pediatric disorder with posture and motor dysfunction in neurological diseases is known as cerebral palsy (CP). Recently, a series of effective techniques have been developed for treatment of CP. These promising methods need high-tech equipment for brain stimulation and mainly classified into invasive and no-invasive approaches. This study aimed to introduce these techniques for treatment of patients who suffer from CP. The potential and performance of currently available brain stimulation techniques have been mentioned in detail. Moreover, the clinical application, safety, efficacy and challenges of these methods have been discussed. Here we review the recent advances in the CP treatment with an emphasis on brain stimulation techniques

## Introduction

A common pediatric disorder with posture and motor dysfunction in neurological diseases is known as cerebral palsy (CP) (1-3). Neurological disorders commonly appeared in early stage of human life statically reported about 3 to 4 cases in 1000 newborn (4, 5). These patients mainly suffer from other problems like orthopedically disorders followed by neurological dysfunctions, unfortunately, that affects their normal life (1). Around 50% of these patients show cognitive deficits. Furthermore, one-third of children suffer from seizure attacks (6, 7). Nowadays, advanced technologies in the field of brain imaging and stimulation have been introduced to medicine for diagnosis and treatment. In addition, cellular therapy methods associated with novel advanced techniques can be useful for treating these disabilities (8-15). 

The aim of this review is to propose effectiveness, profits, and detriments of brain stimulation techniques in CP. This paper runs over the various research efforts within this paradigm reported to date and attempts a selection of the appropriate investigation in this field that are shown in Table 1. Brain stimulation can play a role in remedying CP. Brain tissue can be directly stimulated with electricity. In these non-invasive techniques electrodes directly put in the target site in the scalp and then magnetic field created in the head using magnetic circles (9). We aimed to introduce all advanced techniques clinically used for brain stimulation in neurological disorders such as CP. 

**Invasive procedure for brain stimulation**

**Deep brain stimulation (DBS)**

In this technique, precise electrical impulses directly conducted into the defect area using quad polar electrodes implanted into the brain. Electrical stimulation by this method modulates cellular functions via cell-cell signaling and communication and signaling molecules and chemical mediators. Currently available DBS devices in the market composed of pulse generator set and two electrode leads. Pulse generator set usually fixes in sub-clavicular region adjusted externally and two electrode leads fix in the brain (16-18). This devise act similar to cardiac pacemaker with stimulating and inhibiting activity (16). This adjustment was managed with patient symptoms. The DBS device can be used for improving diatonic and tremor symptoms in Parkinson disorders. Additionally, this approach has been recommended for obsessive-compulsive disease. The adverse sign of neurodegenerative disorders diminishes following the application of this procedure. On the other hand, DBS is significantly better than the ablative surgery via safety and efficacy (17). 

Chronic pain was decreased using DBS for thalamic stimulation (19, 20). Moreover, group of scientists worked on decrease of tremor by thalamic stimulation. DBS had effective impact on tremor and pain (21, 22). In addition, application of this technique on subthalamic nucleus (STN) significantly decreased bradykinesia, tremor and rigidity (23, 24). The satisfied results have been described for the use of DBS in hippocampus and STN for treatment of epilepsy (25). In recent years, high percent of children with dystonia had effective response to this therapeutic method (15, 26). In 2008, a study described the effect of DBS on patient with secondary dystonia. The symptom of disease was improved following using the DBS method. Therefore, this method has been recommended for treatment of various forms of dystonia (27, 28). Although the issue of DBS application for child has received critical attention, but it is in quiet in early stage. 

The last decades have shown a growing trend toward using DBS technique for dystonia complaints in pediatric population (6). The healing potential of DBS was investigated in secondary dystonia associated with CP. Globus pallidus stimulation by DBS method promoted the movement disabilities and dyskinesia in comparison with patients treated with traditional methods such as drug therapy (16). In addition, bilateral pallial DBS could be an alternative method for patients who suffer from dystonia-chorea and CP (29).

The optimum therapeutic spot in these group of sick people is the posterior lateroventral region of Globus pallidus internus (GPi). Diffusion of the stimulation to adjacent structures (mainly Globus pallidus externus), may bring out the little recovery (30). DBS can offer significant changes in multiple domains of general health, dysfunctions and disabilities. Thus, the sequential assessments to evaluate the clinical utilities following DBS via rating scales particularly in children with CP are obligatory (26, 31, 32).

One of the main obstacles faced by many experiments is superficial or deep hematoma after DBS treatment. Additionally, local infection and erosion have existed as a health problem caused by DBS intervention. Another major issue that can impair medical impediment is the digging the electrodes from the skull to the trunk to insert the device in the chest (17).

**Table 1:** A brief summary of the recent papers on brain stimulation

**Author (date)**	**Sample **	**Method**	**Results**
Warren et al (2009)	Child with secondary dystonia	DBS	Improvement of motor function
Vidailhet et al (2009)	Child with dystonia-choreathesosis CP	Bilateral pallidal-DBS	Improvement of motor
Berweck et al (2009)	Child with dystonia-choreathesosis CP	Bilateral pallidal-DBS	Improvement of motor function
Young et al (2013)	11 patients with dystonia aged 7-19 yrs	Cathodal TDCS, over motor cortex at C_3_ or C_4_, 1 ma for 18 min with a 20 min pause interval	Reduction of involuntary over flow activity in a subset of children
Auvichayapat et al (2014)	46 CP patients Aged 8-18 yrs	Anodal TDCS, over left primary motor cortex at M_1_, 1 ma for 20 min on five consecutive days	Sign. Reduction in CP-related spasticity (but not in PROM)
Duarte et al (2014)	24 CP children Aged 5-12 yrs	Anotal TDCS + treadmill training, over primary motor cortex at M_1, _1 ma for 20 min on five weekly sessions for 2 weeks	Sign. Improvements In static balance and functional performance
Grecco et al (2015)	20 children with diparetic CP Aged 5-10 yrs	Anotal TDCS + vitual reality, over primary motor cortex at M_1, _1 ma for 20 min on five weekly sessions for 2 weeks	Sign. Improvement in gait velocity, cadence, gross motor function and independent mobility Sign. Increase in MEP
Gillick et al (2015)	20 patients with hemiparesis Aged 8-21yrs	Ipsilesional anodal and contralesional cathodal TDCS + CIMT, over primary motor cortex at M_1, _0.7 ma for ten 2-hours sessions. TDCS was performed in first 20 min of period.	Sign. Improvement in motor functions
Bhanpuri Et al (2015)	9 patients with dystonia Aged 10-21yrs	Anodal and cathodal TDCS, over motor cortex at C_3_ or C_4_, 2 ma, 9 minutes per day for 5 days	No sign. Changes in dystonia
Bernadette et al (2015)	19 patients with congenital hemiparesis aged 8-17 yrs	5 treatment alternate weekday over 2 weeks rtms for either combined with modified constraint induced-movement therapy(mcimt) or sham rtms combined with mcimt	Minor but not major adverse events such as headaches, cast irritation were found in both experimental and sham groups.
Valle et al (2007)	17 children with spastic CP aged 6-12 yrs quadriparesia	High frequency (5 Hz) rtms, low frequency (1 Hz) rtms , sham rtms over CL ABP motor area, for 5 days	Improvement in elbow movement by high frequency rtms

**Non-invasive procedure for brain stimulation**

**Direct brain Stimulation**

The brain can be provoked with direct method via transcranial fascinated much attention. The past twenty years showed increasingly rapid advances in the field of rehabilitative interventions (33, 34). The direct brain stimulation (TDCS) has more effective than the other approaches for brain stimulation. This method is completely non-invasive and consists of two electrodes, one of them provokes cortical part and the other provokes inhibitory function, to stimuli brain in contact with scalp (35).

Recently an advanced model of TDCS system has been developed and showed some advantages comparing to other methods including safety, cost-effectiveness and portable, permitting neurologist to apply brain stimulation associated with exercise therapy in rehabilitation centers (36-39). The TDCS system targets the regional synaptic in the brain to control the cortical excitability. Not only local cortical stimulation induced by TDCS is not stable, but also it is prompted through weak electrical stimuli to the scalp basically through employment of electrodes (35, 37, 40). A considerable amount of literature have been published on applying the TDCS technique for treatment of neurological disorders (40). Furthermore, this technique has been identified as safe and efficient for pediatric patients. Recently erythematous rash was identified as a side effect in pediatric TDCS treatment (41).

In an original investigation of the efficacy of TDCS treatment on children (between 5 to 12 yr old), the treatment regime was 2 mA for 30 min in 10 sessions. The result of this study represented that children mainly suffered from tingling and itching, mood disorders and also irritability with 28.6%, 42.9% and 36%, respectively (42). The intensity of stimulation was examined in TDCS system. The optimization of stimuli signals via TDCS according to the age of recipient have been recommended especially for children (43). However, research was conducted on child with perinatal ischemic stroke and hemiparesis disorder. Other parameters such as dose play an essential role in child-specific TDCS protocols. In this experiment, magnetic resonance imaging (MRI) was used to evaluate the current flow. They checked some important factors including electrode size and location, dose strength and time period. Computational modeling is necessary to calculate effective dose in order to enhance healing in pediatric stroke through the TDCS guidance (44). Much of available literature on the potential capacity of TDCS technique to increase motor learning in adults and also high clinical studies have reported positive data on motor recovery and reestablishing the equilibrium of the motivation between two hemispheres (45, 46). Case studies on patients containing damaged neurons in hemisphere showed that the TDCS technique can successfully stimulate damaged site in the brain without any side effect on normal tissue. Moreover, many studies have been carried out to restore motor learning disorders thought contralesional stimulation and neuroplasticity pathways (47-49). The mutual impact of anodal TDCS and virtual reality for accelerating walk were evaluated in patient with spastic deformities. In addition, a pilot clinical trial study was deliberate to assess the efficacy of this method in a rehabilitation center. The treated group with both anodal stimulation and virtual reality had satisfied results and improvement. The anodal stimulation together with virtual reality can significantly recover movement in CP patient with spastic di-paretic (26). Combination therapy for example using TDCS with 1 mA strength ended the conquering primary motor cortex and physical training via treadmill for twenty min for 10 d accelerate motor function in patient with spastic di-paretic cerebral palsy. The positive impact of the TDCS technique combined with physical training examined on twenty four patients with spastic diparetic. The temporal function of treated children has been increased (9). The TDCS method associated with physical training augmented some circuits our brain including mediolateral and anterior-posterior. 

This research also established the functional effects of the anodal TDCS technique over primary motor cortex through movement exercise (28). Additionally, the impact of the cathodal TDCS was examined to improve the voluntary activities in patients suffer from the dystonia. Hand function with controlled manner was increased followed by cathode stimulation of healthy hemisphere (50, 51). Moreover, this technique cannot be clinically performed for improving dystonia problem. They carried out a double-blind experiment to assess the impact of the TDCS method on dystonia treatment. The TDCS regime was programmed using 2 mA intensity on cortex for about ten min. The prescribed regime resulted in the symptoms lessening in some patients (51, 52). Gillick et al showed some advantages of the TDCS methods for movement rehabilitation in young and old patient in a control trial study (8, 53). Taken together, many studies and guidelines have been published to validate the efficacy, safety and practicability of the TDCS technique in human. The currently existing data approve the admissibility and the applicability of the TDCS method in CP patient. The published experiments have suggested some signs such as short-lived tingling or slight itching (33, 36, 54). The prescription of this method in CP patient is recommended for many rehabilitation centers.

**Magnetic Stimulation of brain**

In recent years, a novel approach has been introduced in the field of neuroscience known as transcranial magnetic stimulation (55). Abundant studies have been carried out using TMS for investigating neuroplasticity after brain injuries. Magnetic stimulation of brain is a very simple and safe technique. Electromagnetic field is created following TMS induction in the brain tissue (56). In this system, a figure-eight conductive coil was used to conduct electrical current to the neural cells. This magnetic stimulation induces local electrical variations in cortical neural cells based on Faraday's Law. Cortical excitability and eternal modifications in neural behavior can be regulated via monotonous stimulation with TMS (57). However, cortical excitability was assisted or limited via modulation of the intensity of the repetitive TMS (rTMS) pulses (58, 59). 

The main principle of the rTMS is decreasing of the brain function (60) and augments cortical excitability through interhemispheric limitation (61). In early times, Magnetic stimulation of brain was used to facilitate the prediction and improvement after stroke attacks (62, 63) and also neuropsychiatric diseases (64). However, many studies have documented the unknown complications after using the rTMS technique for patients with severe neurological, physiological and psychiatric disorders (64, 65). The application of this system in children is recommended for management of hyperactivity disorder (ADHD) and autism (ASD)(66, 67).

The repetitive TMS has been suggested in high frequency (>5-10 Hz) for treatment of stroke (68-70). Up to now, there is no evidence about any adverse symptoms for rTMS usage (71-73). The use of this advanced technique in grownups (74-77) and patient with stroke are benign and acceptable (78). Both methodological and safety concern are considered as the main obstacle for using rTMS in children. Although it is more effective for movement improvement results in stroke attacks (72, 73, 79, 80), more common disorders in pediatric, treated with rTMS technique, and are pediatric stroke and spasticity (72). The therapeutic effects were tested of the low-frequency rTMS on patient (n=10) suffered from chronic subcortical Arterial Ischemic Stroke (AIS) with motor neuron dysfunction and dyskinesia. In this project patient were divided into two groups, half of them received low-frequency rTMS and the others were considered as a control group. The patients in experiment group were treated with 1200 stimuli for about 7 days. The adverse side effects were not reported in this study and functional improvements, as well as appropriate clinical outcomes, were reported after 7 d follow up post-treatment (78). Cortical stimulation was suggested for children spasticity using the rTMS technique. A study on seventeen patient showed spastic quadriplegia symptoms using repetitive TMS with low and high-frequency intensity (81). rTMS- excited cortical neurons act normally and spasticity improvement can be seen after optimized stimulation(82, 83). Additionally, in 2012 a phase II clinical trial for evaluation of the rTMS technique plus occupational therapy on patients suffered from chronic hemiparesis was conducted (84).

Seizure known as an adverse side effect which was no reported following the use of the rTMS technique for pediatric but headache and local scalp pain were seen in some patient. This method causes transient tinnitus and threshold and further evaluation should be done (85-87). 

The application of this technique has been inhibited for patients having implants in their bodies. 

**In conclusion** the present study provides additional evidence with respect to brain stimulation techniques in patient with neurodegenerative disorders. Neuroscientists reported satisfied results about using advanced technologies including DBS, TDCS, and rTMS for treatment of cerebral stroke, spasticity and patients with abnormal motor function. On the other hand, the safety, efficacy and also no adverse side effects such as seizure following the use of these advanced methods have been discussed in the literature. Further research regarding the role of brain stimulation would be of great help in treatment of neurological disorders. 
